# Spotted-Fever Group *Rickettsia* in *Dermacentor variabilis*, Maryland

**DOI:** 10.3201/eid1008.030882

**Published:** 2004-08

**Authors:** Nicole C. Ammerman, Katherine I. Swanson, Jennifer M. Anderson, Timothy R. Schwartz, Eric C. Seaberg, Gregory E. Glass, Douglas E. Norris

**Affiliations:** * Johns Hopkins Bloomberg School of Public Health, Baltimore, Maryland, USA

**Keywords:** SFG Rickettsia, Dermacentor variabilis, Rickettsia montanensis, Rocky Mountain Spotted Fever, dispatch

## Abstract

Three-hundred ninety-two adult *Dermacentor variabilis* were collected from six Maryland counties during the spring, summer, and fall of 2002. Infection prevalence for spotted fever group *Rickettsia* was 3.8%, as determined by polymerase chain reaction. Single strand conformational polymorphism (SSCP) analysis followed by sequencing indicated that all infections represented a single rickettsial taxon, *Rickettsia montanensis*.

## The Study

Several species of spotted fever group (SFG) rickettsiae have been isolated from ticks in the United States; however, the only species considered to cause human disease in Maryland is *Rickettsia rickettsii*, the causative agent of Rocky Mountain spotted fever (RMSF). The potential pathogenicity of rickettsial organisms is most often predicted by the ability of the species to cause disease in guinea pigs. The reliability of this method has been debated, and researchers have suggested that "every rickettsial species may have pathogenic potential, provided that its reservoir arthropod is capable of biting humans" (1,2).

The prevalence of SFG *Rickettsia* infection in *Dermacentor variabilis*, the primary vector of *R. rickettsii* in the eastern United States, has been estimated in several studies. Prevalences from 0.2% in Ohio (3) to 8.6% in (Table 1) Maryland (4) have been reported. Many studies have implied that these infections were *R. rickettsii*, but few have confirmed these identities (5). Numerous SFG-rickettsial species have been isolated or partially characterized from molecular evidence in the eastern United States; these species include *R. rickettsii*, *R. rhipicephali*, *R. montanensis* (=*R. montana*), *R. parkeri*, and "*R. amblyommi*" (3,6–8). These species have been identified, either together or separately, in areas where RMSF is endemic (Table 2). As the distributions of different SFG-species in disease-endemic areas become better understood, determining the relationship between the rickettsiae involved in human disease and those isolated from vector ticks and mammal and tick reservoirs may be necessary.

**Table 1:** Characteristics of *Dermacentor variabilis* collected in Maryland, 2002^a^

Characteristic	N	% infection with any *Rickettsia* organisms (95% CI)	% infection with SFG *Rickettsia* (%) (95% CI)
All ticks	392	6.1 (4.0–9.0)	3.8 (2.2–6.3)
Sex		p^b^ = 1.000	p = 1.000
Male	185	5.9 (3.0–10.4)	3.8 (1.5–7.6)
Female	207	6.3 (3.3–10.5)	3.9 (1.7–7.4)
County of collection		p = 0.052	p = 0.024
Anne Arundel	1	0 (0–97.5)	0 (0–97.5)
Baltimore	342	6.1 (3.8–9.2)	3.5 (1.8–6.0)
Calvert	17	0 (0–19.5)	0 (0–19.5)
Charles	18	0 (0–18.5)	0 (0–18.5)
Prince George's	1	100 (2.5–100)	100 (2.5–100)
Saint Mary's	13	15.4 (1.9–45.4)	15.4 (1.9–45.4)
Month collected		p = 0.007	p = 0.101
April	146	4.8 (1.9–9.6)	4.8 (1.9–9.6)
May	108	4.6 (1.5–10.5)	1.9 (0.2–6.5)
June	78	2.6 (0.3–9.0)	1.3 (0.03–6.9)
July/August	58	17.2 (8.6–29.4)	8.6 (2.9–19.0)
Unknown^c^	2	0	0

^a^CI, confidence interval; SFG, spotted fever group. ^b^Fisher exact p values. ^c^Ticks with unknown month of collection were excluded from the statistical analyses for this characteristic.

**Table 2:** Univariate odds ratio (OR) associated with any *Rickettsia* organism and with *R. montanensis*

Variable	*Rickettsia* genus–positive			*R. montanensis*–positive		
	OR	95% CI^a^	p value	OR	95% CI	p value
Sex						
Female	1.0	Reference		1.0	Reference	
Male	0.94	0.41–2.16	0.890	0.98	0.35–2.75	0.967
County of collection						
Baltimore	1.02	0.29–3.57	0.969	0.57	0.16–2.09	0.397
All other counties	1.0	Reference		1.0	Reference	
Month collected						
April	1.0	Reference		1.0	Reference	
May	0.96	0.30–3.12	0.951	0.37	0.08–1.84	0.227
June	0.52	0.11–2.58	0.425	0.26	0.03–2.13	0.209
July/August	4.14	1.49–11.47	0.006	1.87	0.57–6.16	0.301

^a^CI, confidence interval.

Differentiating the tick-borne SFG *Rickettsia* before the 1990s depended largely on culture and epitope recognition techniques, such as immunoflourescence and agglutination tests and mouse serotyping with monoclonal antibodies. Genotypic studies of rickettsiae conducted during the 1990s led to two rickettsial genes that can be used to identify rickettsial infections: citrate synthase (*gltA*) and *rOmpA* (9). Citrate synthase encodes the first enzyme of the tricarboxylic acid cycle and is highly conserved among all *Rickettsia* species, serving as a polymerase chain reaction (PCR) target to identify any rickettsial infection. *rOmpA* encodes a surface-expressed protein of SFG-rickettsiae that is important for adhesion to host cells (10). Only SFG *Rickettsia* contain the *rOmpA* gene (11), making it an ideal PCR target to identify SFG *Rickettsia* infections.

Approximately 35 cases of RMSF are reported annually in Maryland. From 1994 through 1998, Maryland ranked 8th nationally, reporting 112 cases. These cases, confirmed by the Maryland Department of Health and Mental Hygiene, meet the Centers for Disease Control and Prevention (CDC) case definition, yet not much information exists to characterize the infection rate of SFG rickettsiae in *D. variabilis* in the state. This cross-sectional study examined the prevalence and composition of SFG *Rickettsia* in *D. variabilis* in Maryland.

In 2002, genomic DNA was extracted from 392 adult *D. variabilis* collected by flagging in Anne Arundel, Baltimore, Calvert, Charles, Prince George's, and St. Mary's Counties, Maryland. Quality of the modified hexadecyltrimethylammonium bromide (CTAB) DNA extractions was verified by amplifying a tick 16S mtDNA fragment (12). Modifying the existing extraction procedure involved an additional phenol:chloroform:isoamyl alcohol (25:24:1) extraction step to further stabilize the extracted DNA. Tick extractions were screened by PCR for evidence of infection with *Rickettsia* by using primers specific to the *Rickettsia* citrate synthase gene (9). The *Rickettsia* infection rate was 6.1% (24/392, 95% confidence interval [CI] 4.0%–9.0%). All *Rickettsia*-positive tick extractions were subsequently screened by PCR for SFG *Rickettsia* by using primers for the *rOmpA* gene of SFG-*Rickettsia* (9). The prevalence of SFG *Rickettsia* infection was 3.8% (15/392, 95% CI 2.2%–6.2%). Single strand conformational polymorphism (SSCP) banding patterns were identical for all tick-derived *rOmpA* PCR amplicons. Similarly, SSCP banding patterns of the tick-derived citrate synthase amplicons for the SFG-*Rickettsia*–positive samples were monomorphic. These results suggest that these tick infections represent a single SFG *Rickettsia* taxon (13). Citrate synthase and *rOmpA* PCR products from three ticks were sequenced with the citrate synthase and shortened *rOmpA* PCR primers, respectively. Sequences of each respective gene fragment derived from these ticks were identical and confirm the SSCP findings (GenBank accession no.: *gltA*, AY548828–AY548830, *rOmpA*, AY543681–AY543683). The derived sequences were also compared to rickettsiae sequences in the public domain and were identical to those derived from *R. montanensis* from *D. andersoni* (GenBank accession no. RMU55823 *rOmpA* and RMU74756 *gltA*).

Prevalence estimates were reported as percentages with exact 95% CI based on the binomial distribution. Fisher exact test was used to compare infection prevalence across the strata of selected characteristics. The association between each characteristic and the prevalence of infection was quantified as odds ratios (OR), calculated with logistic regression or exact methods for categorical data when the data were highly unbalanced. All statistical analyses were performed with STATA (version 7.0; Stata Corporation, College Station, TX) or StatXact (version 5.0.3; Cytel Software Corporation, Cambridge, MA).

The variation in prevalence of *Rickettsia*-positive ticks across all counties was marginally significant (p = 0.052), with a higher prevalence in St. Mary's County compared to all other counties (OR 5.1, 95% CI 0.5–27.2, p value = 0.08). However, only 13 ticks were collected from St. Mary's County, so this estimate was based on limited data. In contrast to the equivocal results for the geographic distribution of *Rickettsia*-positive ticks, temporal heterogeneity was evident, as the prevalence of *Rickettsia*-positive ticks varied significantly with month of collection (p = 0.007). Risk for infection was significantly elevated for any *Rickettsia* organism in ticks collected in July or August (OR 4.1, 95% CI 1.5–11.5) compared to those collected in April. Further analyses combining the data from the spring and early summer months showed that the risk for infection with any *Rickettsia* organism in July or August was even higher (OR 4.7, 95% CI 2.0–11.3). The risk for infection with *R. montanensis* with the late summer months, compared to the spring and early summer months, was somewhat less but still approached statistical significance (OR 3.0, 95% CI 0.8–10.2, p value = 0.06). This observation may be an artifact of diminishing tick abundance later in the summer months.

## Conclusions

The prevalence of SFG *Rickettsia* in *D. variabilis* estimated from this study (3.8%) was lower than that in previous reports from Maryland. However, in regions where RMSF is observed annually, prevalence estimates range widely, from 2% in Connecticut to 10% in Alabama, with intermediate prevalences in New York, Kentucky, Tennessee, and Arkansas (5). In addition, *R. montanensis* had not been previously recognized in Maryland. Most earlier studies of SFG *Rickettsia* infection prevalence did not identify the *Rickettsia* to the species level, although the SFG-positive samples were sometimes assumed to represent *R. rickettsii*. One study in Maryland in which 26 *Rickettsia* isolates were obtained from *D. variabilis* determined the species composition of the rickettsiae. Two isolates were *R. rickettsii*, 1 isolate was *R. bellii* (non-SFG), and 23 (88%) were identified as WB-8-2, a then-unnamed SFG-*Rickettsia* (5). Weller et al. performed a phylogenetic analysis and found WB-8-2 ("*R. amblyommii*") to be closely related to *R. montanensis* (14), although they can be differentiated by serotyping.

*R. montanensis* has been isolated from ticks in other eastern states. During the 1980s, Feng et al. reported that *R. montanensis* represented 41 (91%) of 45 of the SFG isolates from *D. variabilis* collected in Cape Cod, Massachusetts (7). Anderson et al. reported isolation of *R. montanensis* from *D. variabilis* in Connecticut (6), and in 1990, Pretzman et al. reported that most SFG *Rickettsia* isolated from *Dermacentor* ticks throughout Ohio was *R. montanensis* (3). Further, these researchers noted that *R. rickettsii* were not isolated from ticks collected in several Ohio counties where RMSF was considered endemic. These studies illustrate that the rickettsial composition and dynamics within the RMSF-endemic areas are complex and need to be addressed with greater scrutiny.

The role of SFG *Rickettsia* in human health is largely unknown, and many are considered to be nonpathogenic either because the bacteria have not been isolated from humans or they do not demonstrate pathogenicity in animal models. For example, *R. montanensis* is avirulent in guinea pigs but virulent in voles (15). These findings have led to caution when labeling rickettsiae as nonpathogenic (2). *R. montanensis* and other "nonpathogenic" SFG *Rickettsia*–infected ticks may also benefit human health by decreasing *R. rickettsii* in tick populations as a result of the "interference" phenomenon (15).

The findings of this study and others raise important questions. In 2000, a total of 495 cases of RMSF were reported to CDC and 4 deaths were attributed to spotted fever caused by *Rickettsia rickettsii*. The extent to which *R. rickettsii* is the agent responsible for reported cases of RMSF should be reevaluated, considering the number of studies completed in RMSF-endemic regions, including this one, that have found non–*R. rickettsii* as the predominant or only detectable SFG *Rickettsia*.

This research was supported in part by a Cooperative Agreement Award to D.E.N. (U50/CCU319554) and NIEHS training awards (T32ES07141) to J.M.A. and K.I.S.
